# Protective effects of Tadalafil and darbepoetin against ischemia - reperfusion injury in a rat testicular torsion model

**DOI:** 10.1590/S1677-5538.IBJU.2018.0200

**Published:** 2018

**Authors:** Caglar Yildirim, Ozgur H. Yuksel, Ahmet Urkmez, Aytac Sahin, Adnan Somay, Ayhan Verit

**Affiliations:** 1 Department of Urology, Fatih Sultan Mehmet Research & Training Hospital, University of Health Sciences, Istanbul, Turkey; 2 Department of Urology, Haydarpasa Numune Research & Training Hospital, University of Health Sciences, Istanbul, Turkey; 3 Department of Pathology, Fatih Sultan Mehmet Research & Training Hospital, University of Health Sciences, Istanbul, Turkey

**Keywords:** Darbepoetin alfa, Tadalafil, Reperfusion Injury

## Abstract

**Objectives:**

To evaluate protective effects of darbepoetin and tadalafil against ischemia-reperfusion injury in ipsilateral and contralateral testicle.

**Materials and Methods:**

Thirty 3-month-old adult male Wistar-Albino rats were randomly divided into 5 groups (A-E). Sham operation was performed in the first group. In Group B, rats did not received any medication after creating 720 degrees torsion of the left testis. The rats in Group C, D and E received darbepoetin, tadalafil, and darbepoetin/tadalafil combination 30 minutes after creating 720 degrees torsion of the left testis, respectively. The testes of rats in these three groups were detorsioned at 90 minutes after drug administration. Both testes were removed at 30 minutes after detorsion.

**Results:**

There were significant differences between the groups in terms of the degree of histopathological damage, Johnsen score, fibrosis score and caspase-3 immunoreactivity in the torsioned testes (p: 0.000). The results for each parameter in the left testes were significantly better in the darbepoetin / tadalafil combination group. Similarly, there were also significant differences in the contralateral testes (p: 0.000).

**Conclusion:**

The active substances darbepoetin and tadalafil that were used as a combination had protective effects on both testes and produced out better results in preserving testicular histology. Especially in cases where it is not possible to rescue the torsioned testis, this result was more noticeable in the contralateral testis.

## Introduction

Testicular torsion is commonly observed among males in childhood and adolescence. Early diagnosis and treatment of this acute pathology is required as soon as possible. Its incidence rate among males younger than 25 years is 1 / 4000 (1). In spite of a successful surgical intervention, testicular atrophy and, in following years, infertility can develop among 40-60% of the patients (2). Although the first pathology is tissue ischemia, ischemia / reperfusion caused by testicular torsion-detorsion further contributes to the injury. During ischemia, germ cell loss occurs because of low levels of oxygen in proportion to metabolic requirements, decline in cellular energy sources and accumulation of toxic metabolites (3). During the reperfusion phase, a substantial increase in both reactive oxygen radicals (ROS) and reactive nitrogen species (RNS) occurs (4). These free radicals result in an increase in membrane permeability or disruption of membrane integrity through peroxidation of lipids in mitochondria and cell membrane, and cause damage to proteins, enzymes and therefore, the DNA. As a result, germ cell damage caused by the ischemia increases further. While the pathophysiologic process is clearer in the torsioned testis, the tissue damage in the contralateral testis is currently an object of interest. Elucidation of apoptosis mechanisms in the intact testis may facilitate dealing with infertility related to testicular torsion (5). Caspase family, in other words, cysteinyl aspartate-specific proteases, are important signaling molecules, which have various roles depending on the subtype and the organ they are located. The activation level of caspases is used as an indicator of cellular damage in acute ischemic pathologies such as stroke and myocardial infarction (6). Because it is considered that these molecules take part as leverage points in apoptosis of the healthy cell, several molecular researches are conducted about caspases. The role of caspase-3 in this family is particularly important. This protease has been associated with the induction of death cascade and has been accepted as the effector of this point. As it is known that cells gradually go into apoptosis over time with oxidative stress, it is thought that the measurement caspase-3 activation in the tissue can be used to detect apoptotic pathway. The protective roles of numerous therapeutic agents have been shown in organs that sustained I / R injury. However, the studies conducted to date have shown that tissue damage caused by torsion cannot be fully prevented and there is currently no known therapeutic agent that could be used in clinical practice. On the other hand, although testis is the most easily torsioned organ in the body, there is no satisfactory clinical research in the literature regarding this subject. For this reason, studies using experimental models still continue regarding the treatment of testicular torsion. Tadalafil (Td) is a phosphodiesterase-5 inhibitor (PDE5i) commonly used in the treatment of erectile dysfunction in current urology practice (7). The inhibition of phosphodiesterase (PDE) enzymes increases cGMP levels in the tissue, therefore resulting in relaxation of smooth muscles. There are several studies in the literature investigating the I / R injury in testicular in rat testicular torsion model using different PDE5 inhibitors such as sildenafil and vardenafil (8). Erythropoietin (EPO), on the other hand, is produced by the kidneys in reaction to tissue hypoxia, and it increases the amount of mature erythrocytes in the circulation by stimulating erythroid progenitor cells via erythropoietin receptors (EPO-R) and increases the oxygen-carrying capacity. In recent years, functional EPO-R receptors have been shown to exist in cells and organs, which do not take part in erythropoiesis (9). In most of the experimental studies conducted on animals, it was discovered that EPO possesses a potential role as a multifunctional endogenic mediator that provides a protective effect against I / R injury in various tissues and organs (10). Darbepoetin (DPO), which is a new recombinant erythropoietic protein, is a long-acting EPO analogue. It has been successfully used in the treatment of various types of anemia even if they have not been caused by EPO deficiency in their etiology, as it increases the oxygen-carrying capacity.

In light of this information, the present study aimed at investigating the protective effect of DPO, Td and combination of these two agents on both testes in experimentally induced I / R model in rats.

## MATERIALS

### Laboratory animals

In our study, a total of thirty male 3-month-old adult Wistar-Albino rats weighing 200-300 grams were used. During the experiment, the rats were given ad libitum access to water and bait. The rats were maintained in groups inside the laboratory under normal room temperature (22°C).

### Drugs

Ketamine: 50-mg / kg ketamine (Ketalar^®^, Pfizer Pharma GMBH, Germany), administered intraperitoneally (IP) for the purpose of anesthesia.

Xylazine hydrochloride: 10-mg / kg xylazine hydrochloride (Alfazyne^®^, 2%, Alfasan International, 3440 AB, Woerden, Holland), administered via IP injection for anesthesia.

Darbepoetin alpha: 1 mcg / kg darbepoetin alpha (Aranesp ready-to-use syringe, Amgen İlaç), administered via IP injection in the experiment group.

Tadalafil: 0.25 mg / kg tadalafil (Lifta tablet, Abdi İbrahim İlaç Sanayi ve Ticaret A.Ş.), administered via IP injection in the experiment group.

## METHODS

The rats were randomly divided into 5 groups, each comprising 6 rats. Prior to surgical intervention, general anesthesia was induced by intraperitoneal (IP) administration of ketamine 50 mg / kg and xylazine hydrochloride 10 mg /kg. In order to ensure physical inactivity of rats, same drugs were administered at the same dosages when required. After induction of anesthesia and ensuring stabilization, scrotal area was shaved and antisepsis was ensured with 10% povidone iodine solution. Group A was assigned as the sham operation group. Mid-scrotal incision was performed and the left testis and spermatic cord were released. Without creating testicular torsion, the testis was placed into the scrotum and secured in the anatomic position, and the scrotum was primarily closed with 6 / 0 prolene suture. After 120 minutes, both testes were removed and fixed in 10% neutral formaldehyde solution for histopathologic examination. I / R injury was created in Group B and the rats received no medications. The left testis and spermatic cord were released after mid-scrotal incision. An experimental testicular torsion model was created by rotating the left testicle with its cord 720 degrees clockwise, and the testicle was fixed to the scrotum's internal surface with 6 / 0 propylene suture. At 120 minutes after torsion, reperfusion was achieved by detorsion. For the experiment to be as close to the real life situation as possible, both testes were numerated and fixed in 10% neutral formaldehyde solution for histopathologic examination at 30 minutes after detorsion. I/ R model was created and DPO was administered to rats in Group C. Testicular torsion was duly created in the rats. DPO 1 mcg / kg was injected intraperitoneally at 30 minutes after torsion. At 120 minutes after torsion, reperfusion was achieved by detorsion. At 30 minutes after detorsion, both testes were numerated and fixed in 10% neutral formaldehyde solution for histopathologic examination. I / R model was created and Td was administered to the rats in Group D. Testicular torsion was duly created in the rats. At 30 minutes after torsion, 0.25 mg / kg of Td was injected intraperitonally to the rats. At 120 minutes after torsion, reperfusion was achieved by detorsion. At 30 minutes after detorsion, both testes were numerated and fixed in 10% neutral formaldehyde solution for histopathologic examination. I / R model was created and a combination of DPO and Td was administered to the rats in Group E. Testicular torsion model was created using the same method. At 30 minutes after torsion, 1 mcg / kg of DPO and 0.25 mg / kg of Td were injected intraperitoneally. At 120 minutes after torsion, reperfusion was achieved by detorsion. At 30 minutes after detorsion, both testes were numerated and fixed in 10% neutral formaldehyde solution for histopathologic examination. Then, the rats in each group were decapitated under anesthesia via guillotine and the experiment was finalized.

### Histopathologic Analysis

Tissue samples from each rat were fixed in 10% neutral formaldehyde and a sample was obtained by a pathologist from the midsection of the testis as to contain the widest surface. Each sample was taken into tissue processing in one cassette. The tissue dehydration, clearing and infiltration processes were conducted with fully enclosed Leica ASP00 S tissue processing device. Each of the processed tissues was transformed into paraffin tissue blocks with Thermo Shendon Histocentre 3 device. Three slides containing 4-micron-thick sections were prepared from each paraffin block using Thermo Rotary Microtome and one of the slides was stained with hemotoxilin-eosine (H-E) in Sakura Tissue-Tek Film enclosed system stainer and film cover slipper device. Another slide was manually stained using Masson-Trichrome (M-T) with Bio-Optica 04-010802 kit, according to its operating instructions. The third slide was transformed onto polylysine slide and was immunohistochemically stained with Caspase-3 (CPP32, Ab-4; Catalog #RB-1197-R7 Thermo Scientific) in Leica Bond-Max automated immunohistochemistry device. The stained slides were evaluated and scored by a pathologist according to the above tables using the Olympus CX41 Japan light microscope. In addition, each examined section was digitally photographed.

In assessing the histological damage, each seminiferous tubule observed on the cross-section of the H-E stained slide was summed by grading the tubules between 1 and 4 and the scores achieved by dividing the sum to the total number of tubules.

- Grade I: Shows the normal testicular structure and organization of organized germinal cells;
- Grade II: Shows less organized, non-cohesive germinal cells and closely packed seminiferous tubules;
- Grade III: Shows irregular structure containing non-living germinal cells with shrunken pyknotic nuclei and indistinctive seminiferous tubule borders;
- Grade IV: Shows seminiferous tubules tightly surrounded by coagulation necrosis of the germ cells.

For Johnsen score, each seminiferous tubule observed on the cross-section of the H-E stained slide was graded from 1 and 10 and the case score was achieved by dividing to the total number of tubules.

For fibrosis scoring, uptakes on M-T stained slide sections were scored as; (0) none, (1) minimal, (2) mild, (3) moderate and (4) severe.

For Caspase-3, five areas containing at least 1000 germ cells excluding the interstitial cells on immunohistochemically stained slides were examined randomly under light microscope at 400 x magnification. The ratio of germ cells with cytoplasmic and / or nuclear staining in this area were scored as; (0) no staining, (1) 0-1%, (2) > 1-5%, (3) > 5-25% and (4) > 25%.

### Statistical analysis

IBM SPSS Statistics 22 (IBM SPSS, Turkey) program was used for statistical analyses of the study findings. During the evaluation of study data the normality of parameters were assessed via Shapiro Wilks normality test. In the evaluation of the study data, Kruskal Wallis test was used for intergroup comparison of non-normally distributed parameters found in the assessment of quantitative data, and Mann Whitney U test was used to determine the group that caused significant difference. Significance was evaluated at the level of p < 0.05.

## RESULTS

**Figure 1:**
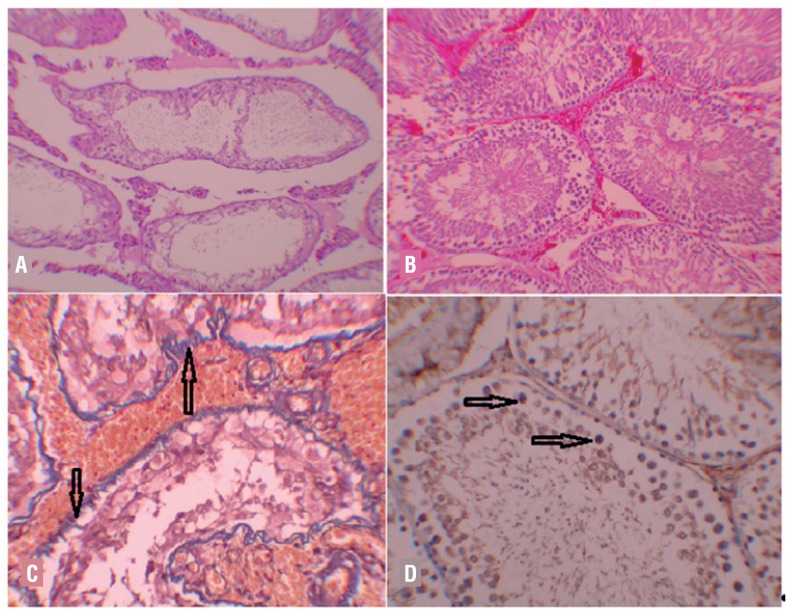
A) I/R group, Left testis: Irregular contours, seminiferous tubules that only contain Sertoli cells and spermatogonia (H-E x100). B) DpO/Td combination Group, Left Testicle: Tubules on a hyperemic background, Johnsen score partially low, but organized tubules (H-E x100). c) I/R Group; Left Testicle M-T: Diffuse fibrosis in basal membrane of deformed seminiferous tubules (arrow)(M-T x200). D) I/R group, caspase-3, Left testicle: Distinctive staining on germ cells (arrow)(IHK x200).

The level of histopathological damage, Johnsen's spermatogenesis scores, fibrosis rates and immunohistochemical tissue caspase-3 levels of both testicles in a total of 30 rats in five groups were detected as shown in the tables (Tables 1 A and B). When the left testicles were compared between groups, there were statistically significant differences in terms of the level of histopathological damage (p: 0.000; p < 0.05). The values of the sham group were significantly lower in comparison to the other groups (p: 0.000; p < 0.05). In addition, the values in the I / R group were significantly higher than the values of the DPO / Td combination group (p: 0.038; p < 0.05) (Figure-1A). There were statistically significant differences between groups in terms of Johnsen score (p: 0.000; p < 0.05). The values of the sham group were significantly higher than the values of the I / R (p: 0.000), DPO (p: 0.000), Td (p: 0.000) and DPO / Td combination (p: 0.035) groups. The Johnsen score values of DPO / Td combination group were significantly better than the values of I/R (p: 0.000) and Td (p: 0.019) groups (Figure-1B). When the fibrosis scores between the groups were compared, there were still statistically significant differences (p: 0.000; p < 0.05). The fibrosis score in the I / R group was significantly higher than the scores in DPO (p: 0.005), Td (p: 0.002) and DPO / Td combination (p: 0.001) groups (Figure-1C). There were also statistically significant differences between the groups in terms of immunohistochemical caspase-3 levels, which is the last parameter compared between the groups. While the values of the sham group were lower than the other groups (p < 0.05), the values in the I / R group were significantly higher than the values of the DPO / Td group (p: 0.006; p < 0.05) (Figure-1D).

**Table 1:** (A and B) - Analysis of study parameters in the left then in the right testis between groups.

Table A				
Group Left	Histopathological damage	Johnsen's score	Fibrosis score	Caspase-3 levels
	Mean ± SD (Median)	Mean ± SD (Median)	Mean ± SD (Median)	Mean ± SD (Median)
A) Sham	1.23 ± 0.07 (1.2)	8.83 ± 0.27 (8.8)	0 ± 0 (0)	0.42 ± 0.51 (0)
B) I / R	2.42 ± 0.91 (2.4)	5.78 ± 1.04 (6.3)	1.17 ± 0.58 (1)	2.42 ± 0.79 (2)
C) DPO	1.96 ± 0.66 (1.8)	7.48 ± 0.7 (7.7)	0.42 ± 0.51 (0)	1.67 ± 0.98 (1.5)
D) Td	2.09 ± 0.64 (2)	7.05 ± 0.79 (7.1)	0.33 ± 0.49 (0)	1.83 ± 0.83 (2)
E) DPO+Td	1.79 ± 0.7 (1.7)	8.06 ± 0.93 (8.2)	0.25 ± 0.45 (0)	1.17 ± 1.03 (1)
p	0.000*	0.000*	0.000*	0000*

Kruskal Wallis Test* p < 0.05

**Table:** Table B

Group Right	Histopathological damage	Johnsen's score	Fibrosis score	Caspase-3 levels
	Mean ± SD (Median)	Mean ± SD (Median)	Mean ± SD (Median)	Mean ± SD (Median)
A) Sham	1 23 ± 0.09 (1.2)	8.88 ± 0.19 (8.8)	0 ± 0 (0)	0.67 ± 0.52 (1)
B) I / R	1.6 ± 0.19 (1.6)	6.61 ± 0.31 (6.6)	0.83 ± 0.41 (1)	1.83 ± 0.41 (2)
C) DPO	1.45 ± 0.13 (1.4)	8.04 ± 0.19 (8)	0 ± 0 (0)	0.83 ± 0.41 (1)
D) Tada	1.52 ± 0.18 (1.5)	7.79 ± 0.23 (7.9)	0 ± 0 (0)	1.17 ± 0.41 (1)
E) DPO + Tada	1.13 ± 0.08 (1.1)	8.88 ± 0.32 (8.9)	0.17 ± 0.41 (0)	0.33 ± 0.52 (0)
p	0.000*	0.000*	0.001*	0.002*

Kruskal Wallis Test* p < 0.05

**Figure 2:**
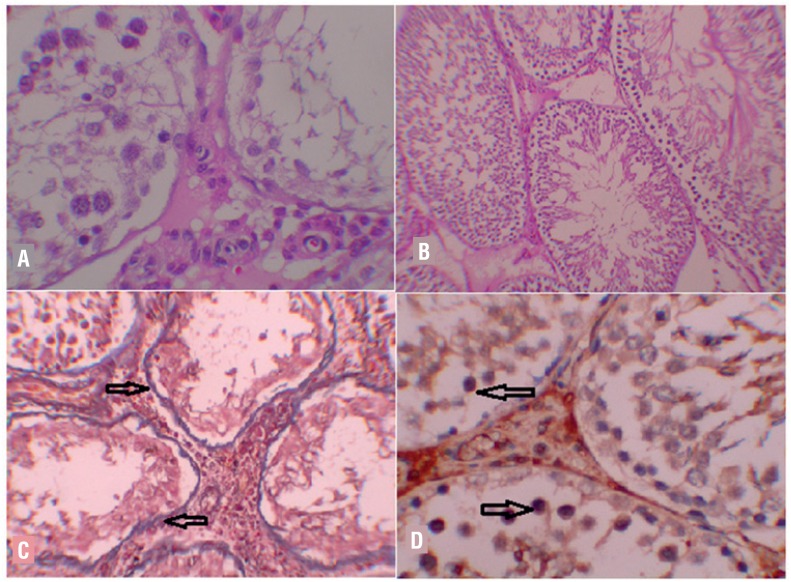
A) I/R group, right testicle: Spermatogenesis arrest at spermatocyte level and seminiferous tubules containing only Sertoli cells (H&E x400). B) DpO/Td combination group, Right Testicle: Regular sized seminiferous tubules with slightly decreased Johnsen score (H&E x100). c) I/R Group; Right Testicle M-T: Severe fibrosis on basal membrane of deformed seminiferous tubules (arrow)(M-T x200). D) I/R group, caspase-3, Right testicle: Staining on several germ cells (arrow)(IHK x200).

All parameters were also compared with the right testis in which testicular torsion was not created. There were statistically significant differences in terms of the degree of histopathological damage (p: 0.000; p < 0.05). While the values in the sham group were lower than the values of the other groups (p < 0.05), DPO / Td combination group values were significantly lower than the I /R, DPO and Td groups (p: 0.004; p < 0.05) (Figure-2A). Similarly, while the Johnsen's spermatogenesis scores were higher in the sham group in comparison to other groups (p: 0.004; p < 0.05), the values of DPO / Td combination group were statistically better than the values of I / R, DPO and Td groups (p: 0.004; p < 0.05) (Figure-2B). Evaluation of tissue fibrosis rates also showed significant differences (p: 0.001; p < 0.05). The fibrosis rate in the I / R group was higher than the fibrosis rates in the sham (p: 0.005), DPO (p: 0.005), Td (p: 0.005) and DPO / Td combination (p: 0.027) groups (p < 0.05) (Figure-2C). There were also significant differences in terms of the last evaluated parameter, caspase-3 immunohistochemistry levels (p: 0.002; p < 0.05). The values in the I / R group were significantly higher than the values in the sham (p: 0.006), DPO (p: 0.006), Td (p: 0.027) and DPO / Td combination (p: 0.004) groups, whereas the values in the Td group were higher than DPO / Td combination group (p: 0.018; p < 0.05) (Figure-2D).

## DISCUSSION

Darbepoetin alpha and tadalafil have already been studied in ischemia - reperfusion injury but as far as we know, our study is the first one in which these both drugs were used together and their combined protective effects against ischemia-reperfusion injury in ipsilateral and contralateral testicle were investigated.

It is known that the torsion / detorsion model which was created experimentally on the testicle is a mode of I / R injury and germ cells are the cell group that is the most sensitive to I /R injury (11, 12). As a result of a set of reactions triggered by the tissue hypoxia and the following oxidative stress caused by reperfusion, these cells undergo an irreversible apoptotic process. A large number of cytokines and mediators take part in pathophysiology of this process. A common signal transduction pathway has been the subject of several studies in the literature in recent years. Although the actual mechanism in the initiation and progression of apoptosis is not known for all caspases, the involvement of caspase enzyme family as a sole indicator and a potential leverage make them the center of interest particularly for new drug investigations. Caspase-3, which was also immunohistochemically measured in our study, is considered an effector in the beginning of death cascade and an important marker for cells undergoing apoptotic signal pathway.

It is reported that in similar studies in the literature using I / R injury model, the damage does not only occur in the testis on which the model has been created but also in the contralateral testicle, and the presence of chemicals that could be used to protect particularly the contralateral testis continues to be the subject of interest. It is without a doubt that the protection of non-torsioned testis plays out a significant role in fertility potential. While higher levels of lipid peroxidation, low antioxidant capacity and high histopathological damage levels are reported for contralateral testicle on some of the conducted studies, there are also opposing studies which claim that no significant damage occurs in the contralateral testis (13, 14). Also in our study, the histopathological damage level, fibrosis rate and caspase-3 immunohistochemistry levels of contralateral testes, especially of those in the I / R group, have been found to be significantly higher than in the sham group; in addition, also the spermatogenesis level in these testes were significantly lower than the sham group. Autoimmunization against spermatogonium, disruption of blood-testis barrier, decrease in tissue perfusion by reflexive increase in the sympathetic response and increase in the production of ROS can be implicated in the pathophysiology of the damage that occurred in the contralateral testis (15). It is another interesting point that the death of cells in the non-torsioned side occurs not through pathological necrosis but rather through caspase pathway, which is an indicator of apoptosis. The results of our study can also be explained by similar mechanisms.

Effectiveness of several different chemical agents for minimizing the germ cell damage and contribution of torsion to infertility after an early detorsion process following the testicular torsion have been investigated in numerous studies; however, a very few of these have been introduced into clinical practice due to their tolerable side effect profiles (16-18). In recent years, studies on tadalafil have been added to earlier studies using I / R injury damage model where sildenafil, vardanafil and other PDE5 inhibitors have been used. The results of these studies are contradictory in general terms. In one of these studies, it has been claimed that the use of Td combined with arginine could be effective in protecting both testes from I / R injury (13). Beheshtian et al. make reference to the protective effects of sildenafil administered prior to detorsion and they based this finding upon the increased antioxidant enzyme levels, decreased lipid peroxidation and germ cell apoptosis they have found in their experiment (17). Similarly, there are studies showing that the use of low-dose sildenafil could contribute to the protection of contralateral testis and better histopathological results could be achieved by administration of vardanafil at 30 minutes after torsion (8, 19). In contrast, there are publications reporting that sildenafil and vardanafil have not protective effect after 1 hour of ischemia followed by 2 hours of reperfusion (20). EPO has also been used in similar experimental models. DPO, which is a recombinant analogue of EPO, is a more useful agent due to its easier administration and its long-term effects. There are several studies in the contemporary literature regarding EPO, which main mechanism is increasing the capacity of carrying oxygen to tissues by increasing the amount of mature erythrocytes in the circulation. In some of those studies, it was shown that EPO receptors to which EPO binds to exerts its effect are not only found in erythroid progenitor cells but also in tissues with no erythropoiesis task such as brain, spinal cord, heart and the testis (21, 22). In addition, it is known that EPO contains antiapoptotic, antioxidant, anti-inflammatory and angiogenic effects (23). From this point of view, the use of EPO for protective purposes in I / R injury models where hypoxia takes the main part has been one of the major focuses of our research. In some contemporary publications, it is reported that EPO exerts a protective effect on spinal cord, eye, intestine, lungs and liver damages caused by I / R injury (24-26). The mechanism of this protective effect is yet to be elucidated. However, it is considered that the receptor-related tyrosine kinase pathway is the main intracellular pathway in hematopoiesis and neuroprotection (27). Moreover, Abdelrahmanet et al. have provided evidence that protecting the tissues with EPO that are exposed to oxidative stress is secondary to inhibition of caspase-3, 8 and 9 and hence the antiapoptotic effect of EPO (28). In our study, the histological damage levels, Johnsen spermatogenesis scores, fibrosis rates and caspase-3 immunohistochemistry levels of both testicles in the experimental group where DPO is used in combination with tadalafil were significantly better than the other experimental groups.

## CONCLUSIONS

It is known from the experiments conducted on animals that acute unilateral testicular torsion bears the potential to negatively affect both testes and as a result, to cause infertility because of the impaired spermatogenesis. In our study, the active substances darbepoetin and tadalafil that were used as a combination have protective effect on both testes. The combined use of these two molecules results in better protection of testicular histology, better spermatogenesis results, less fibrosis and less caspase-3 accumulation in the tissue. Especially in cases where it is not possible to save the torsioned testis, this result was more noticeable in the contralateral testis, which is vital for the protection of fertility potential; in our study, it was observed that the contralateral testis is affected by this pathology, and this effect can be minimized with treatment. However, it is a fact that for the analysis of usage of these molecules in clinical practice and to obtain more accurate and reliable results, there is a need for further animal experiments as this study comprises small sample of animals.

And also there is a need for further clinical studies in addition to the animal experiments.
